# Lactoferrin Induces Osteoblast Growth through IGF-1R

**DOI:** 10.1155/2015/282806

**Published:** 2015-07-28

**Authors:** Jian-Ming Hou, En-Yu Chen, Fan Lin, Qing-Ming Lin, Ying Xue, Xu-Hua Lan, Man Wu

**Affiliations:** ^1^Endocrinology Department, Fujian Provincial Hospital, No. 134 Dong Jie Road, Fuzhou, Fujian 350001, China; ^2^Provincial Clinical Medical College of Fujian Medical University, No. 134 Dong Jie Road, Fuzhou, Fujian 350001, China

## Abstract

*Objectives.* To investigate the role of the IGF-1R by which lactoferrin induces osteoblast growth. *Methods.* Osteoblast received 5 d lactoferrin intervention at a concentration of 0.1, 1, 10, 100, and 1000 *μ*g/mL, and the IGF-1 and IGF-1R were detected using RT-PCR and western blot. The osteoblast into the control, 100 *μ*g/mL lactoferrin, Neo-scramble (NS, empty vector), NS + 100 *μ*g/mL lactoferrin, shIGF-1R and shIGF-1R + 100 *μ*g/mL lactoferrin group. We test the apoptosis and proliferation and the level of PI3K and RAS in osteoblasts after 5 d intervention. *Results.* (1) 1, 10, 100, and 1000 *μ*g/mL lactoferrin induced the expression of IGF-1 mRNA and protein. 10 *μ*g/mL and 100 *μ*g/mL lactoferrin induced the expression of IGF-1R mRNA and protein. (2) Lactoferrin (100 *μ*g/mL) induced osteoblast proliferation while inhibiting apoptosis. Osteoblasts with silenced IGF-1R exhibited decreased proliferation but increased apoptosis. MMT staining and flow cytometry both indicated that there was no significant difference between the shIGF-1R group and the shIGF-1R + 100 *μ*g/mL lactoferrin group. (3) Lactoferrin (100 *μ*g/mL) induced PI3K and RAS phosphorylation and silence of IGF-1R resulted in decreased p-PI3K and p-RAS expression. Lactoferrin-treated shIGF-1R cells showed significantly higher level of p-PI3K and p-RAS when compared with shIGF-1R. *Conclusion.* Lactoferrin induced IGF-1/IGF-1R in a concentration-dependent manner. Lactoferrin promoted osteoblast proliferation while inhibiting apoptosis through IGF-1R. Lactoferrin activated PI3K and RAS phosphorylation via an IGF-1R independent pathway.

## 1. Introduction

Lactoferrin, the transferrin family member, is an iron-binding glycoprotein that displays anti-inflammatory, antibacterial, and immunomodulatory activities [1]. Lactoferrin can be secreted by exocrine epithelial cells, and its concentration in normal human serum varies within the range of 2–7 × 10^−6 ^g/mL [2]. Serum lactoferrin mainly derives from neutrophils, and its local concentration may increase during inflammation. Our previous studies demonstrated that injection of lactoferrin at a concentration of 1 g·kg^−1^·d^−1^ and 2 g·kg^−1^·d^−1^ significantly increased bone mass and improved bone microstructure in ovariectomized rats [3].* In vitro* studies also revealed that lactoferrin could in one aspect induce osteoblast proliferation and differentiation, while in another aspect inhibit osteoblast apoptosis. However, the molecular mechanisms by which lactoferrin regulates osteoblast growth are still unclear [4]. A specific receptor for lactoferrin has been cloned from human intestine [5], but the expression of receptor mRNA could not be detected in osteoblast, and the receptor was not expressed on the surface of any lactoferrin target cells. Present study suggested that osteoblast expressed low-density lipoprotein receptor-related protein LRP1 and LRP2 on its surface, and lactoferrin could activate the P42/44/MAPK signaling pathway through interaction with LRP1, indicating that LRP1 at least partially participated in the proliferation of osteoblast [6]. Lactoferrin could also induce osteoblast proliferation by activating PI3K, but the mechanisms were yet to be clarified. Our early study has found there was no statistical difference between the group in the presence of 5 *μ*M OSI906 (the selective inhibitor of IGF-1 receptor and insulin receptor) and LF and the group only exposed to 5 *μ*M OSI906. It indicated that OSI906 could block the mitogenic effect of LF in osteoblasts [7]. So in this study, we design the shIGF-1R to verify that lactoferrin promotes osteoblast growth by IGF-1R receptor; we silenced the insulin-like growth factor-1 receptor (IGF-1R) in osteoblast and detected the level of proteins involved in downstream signaling pathways and thereby investigated the role of the IGF-1R by which lactoferrin induces osteoblast growth.

## 2. Materials and Methods

### 2.1. Cell Culture

Eight Sprague-Dawley male rats aged 24 h were killed by cervical dislocation. Rats' heads were obtained under sterilized condition, and the skulls were sampled in a PBS-filled petri dish. After removal of connective tissues by PBS washing, skulls were cut into a volume of approximately 1 mm^3^, digested subsequently by 0.25% trypsin (Hyclone, USA) and 0.1% type I collagenase (Invitrogen, USA) and then inoculated in a 25 cm^2^ flask for cell culture and passage in a 37°C incubator containing 5% CO_2_. Afterwards, osteoblast cells (passage 3) were seeded on a 6-well plate at a concentration of 1 × 10^5^ cells/well for adherent growth, and were synchronized by culturing in serum-free DMEM media for 24 h. Cells were randomly assigned into a control group and 5 experimental groups, which, respectively, received lactoferrin (New Zealand, purity > 90%) intervention at a concentration of 0.1 *μ*g/mL, 1 *μ*g/mL, 10 *μ*g/mL, 100 *μ*g/mL, and 1000 *μ*g/mL for 5 consecutive days.

### 2.2. Real-Time PCR

Total cellular RNA was extracted by Trizol reagent (Invitrogen, USA), and reverse transcription (20 *μ*L system) was performed according to the instructions provided by the kit. RT-PCR was performed using the SYBR Premix Ex Taq TM II kit (DRR081A, Takara, Japan) on a Thermal Cycler Dice TM Real Time system (TP800, Takara, Japan). Primers for *β*-actin and IGF-1 were designed and synthesized by Takara (Table 1). Quantitative gene expression analysis was carried out by using the 2^−ΔΔCt^ method.

### 2.3. Western Blot

Total cellular protein was extracted by a protein extraction kit (Newgene Bio, Shanghai, China) and quantified by a BCA protein assay kit (Newgene Bio, Shanghai, China). After that, a total of 60 *μ*g protein was loaded and electrophoresed through a 10% SDS-PAGE gel. Separated proteins were subsequently transferred onto a PVDF membrane and incubated with primary antibody (Abcam, America) at 4°C overnight, then with rabbit anti-mouse secondary antibody (Zhongshan Biotech, Beijing, China) at room temperature for 2 h. Western blots were developed using the SuperSignal West Dura Extended Duration Substrate, and image analysis was carried out after X-ray scanning. The experiment was repeated 3 times.

### 2.4. Lentiviral Transfection

Based on the principle of shRNA design and according to the sequence of IGF-1R, 3 shRNA sequences were designed and synthesized by Shanghai Newgenebio Company (Table 2). The shRNA vectors were cotransfected into HEK293T cells with the lentiviral packaging plasmids, and the recombinant lentiviral particles were used to infect primary rat osteoblasts. RT-PCR was performed to verify the efficiency of gene silencing by detecting IGF-1R expression, and the shRNA vector exhibiting the highest silencing efficiency (S1, silencing efficiency > 80%) was selected for future experiment. Noninfected and infected osteoblasts were digested in logarithmic growth phase, and were, respectively, inoculated in a 6-well plate at a concentration of 1 × 10^5^ cells/well. Both noninfected and infected cells were then randomly assigned into a control group and 5 experimental groups, including 100 *μ*g/mL lactoferrin group, Neo-scramble (NS, empty vector) group, NS + 100 *μ*g/mL lactoferrin group, shIGF-1R transinfection group, and shIGF-1R + 100 *μ*g/mL transinfection group. Cells were then cultured in a 37°C incubator containing 5% CO_2_.

### 2.5. Determination of Cell Proliferation

Cells obtained from the transfection experiment were inoculated in a 96-well plate at a concentration of 3 × 10^3^cells/well. Each group had 6 repeats. After 5 d of lactoferrin intervention, cells were added with 20 *μ*L MTT (5 mg/mL, Gibco) and cultured for 4 h. After that, 150 *μ*L of dimethyl sulfoxide (DMSO, Sigma) was added to each well, and the plate was oscillated for 10 min until the crystals were fully dissolved. Optical density (OD) at a wavelength of 490 nm was determined by a microplate reader, and a blank control was introduced. The experiment was repeated 3 times.

### 2.6. Determination of Cell Apoptosis

Cells obtained from the transfection experiment were cultured in an incubator (5% CO_2_, 37°C) for 24 h and then digested by trypsin. Each group had 3 repeats. After washing with ice-cold PBS, cells were resuspended in 1x Binding buffer to a concentration of 1 × 10^6^ cells/mL. For detection of apoptosis, 5 *μ*L of 7-AAD and 5 *μ*L of annexin V-APC (Nanjing KGI) were added to 500 *μ*L of cell suspension and incubated for 10 minutes at 4°C in the dark. Cell apoptosis was tested by a flow cytometry within 1 h.

### 2.7. Detection of IGF-1R Downstream Signaling in Osteoblast

Cells obtained from the transfection experiment were cultured in a 37°C incubator containing 5% CO_2_. Each group had 3 repeats. After 5 d of lactoferrin intervention, osteoblast were digested with trypsin and collected for western blot detections of PI3K (CST #4292), p-PI3K (CST #4228), RAS (Santa Cruz SC-863), and p-RAS (CST #3321).

### 2.8. Statistical Analysis

Experimental data were presented as mean ± SD. Software SPSS16.0 was used for one-way analysis of variance (ANOVA). *P* < 0.05 was considered as significant difference.

## 3. Results

### 3.1. The Effects of Lactoferrin on IGF-1/IGF-1R mRNA Expression in Osteoblast

**Figure 1:**
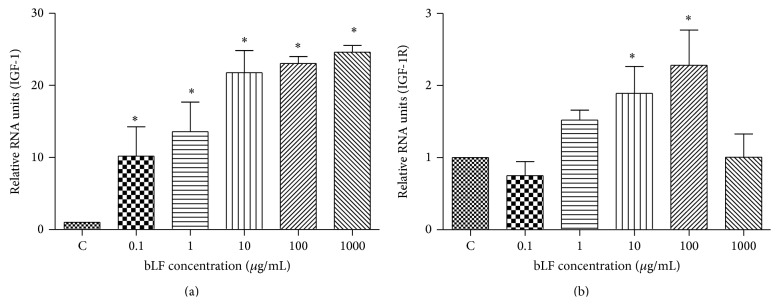
The effects of lactoferrin on IGF-1/IGF-1R mRNA expression in osteoblast. (a) IGF-1, (b) IGF-1R; ^*^ *P* < 0.01 compared with control. The experiment was repeated 3 times.

Lactoferrin intervention significantly increased (*P* < 0.01) IGF-1 mRNA expression in a concentration dependent manner, and osteoblasts added with 1000 *μ*g/mL lactoferrin showed the highest level of IGF-1 mRNA expression. Lactoferrin intervention at a concentration of 10 *μ*g/mL and 100 *μ*g/mL significantly (*P* < 0.01) induced IGF-1R mRNA expression, but lactoferrin with other concentrations (0.1 *μ*g/mL, 1 *μ*g/mL, and 1000 *μ*g/mL) had no significant influence on IGF-1R mRNA expression (Figure 1).

### 3.2. The Effects of Lactoferrin on IGF-1/IGF-1R Expression in Osteoblast

**Figure 2:**
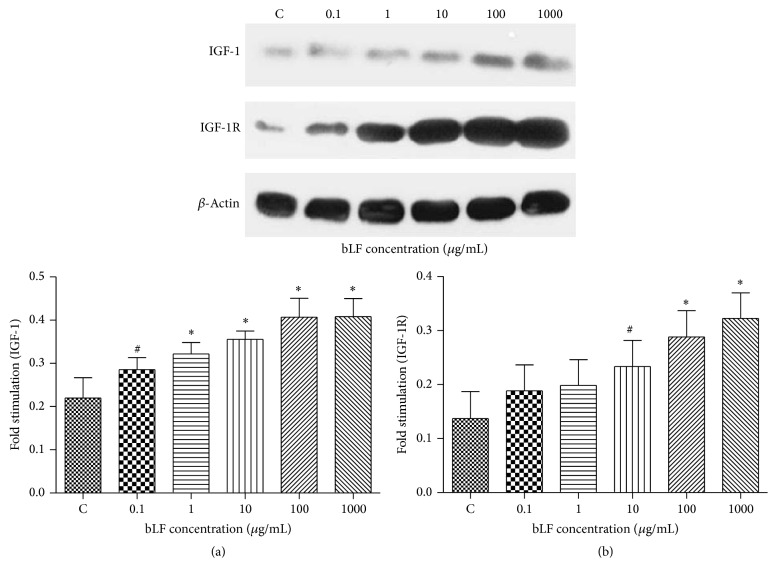
The effects of lactoferrin on IGF-1/IGF-1R protein expression in osteoblast. (a) IGF-1, (b) IGF-1R; ^#^ *P* < 0.05 and ^*^ *P* < 0.01 compared with control. The experiment was repeated 3 times.

Lactoferrin intervention significantly induced (*P* < 0.01) IGF1 expression in all osteoblasts except those added with 0.1 *μ*g/mL lactoferrin (*P* < 0.05). Lactoferrin intervention at a concentration of 10 *μ*g/mL (*P* < 0.05), 100 *μ*g/mL (*P* < 0.01), and 1000 *μ*g/mL (*P* < 0.01) significantly increased IGF1R expression, while cells in other groups showed no significant variations in IGF1R expression (Figure 2).

### 3.3. Lentiviral Vector Construction and Selection

**Figure 3:**
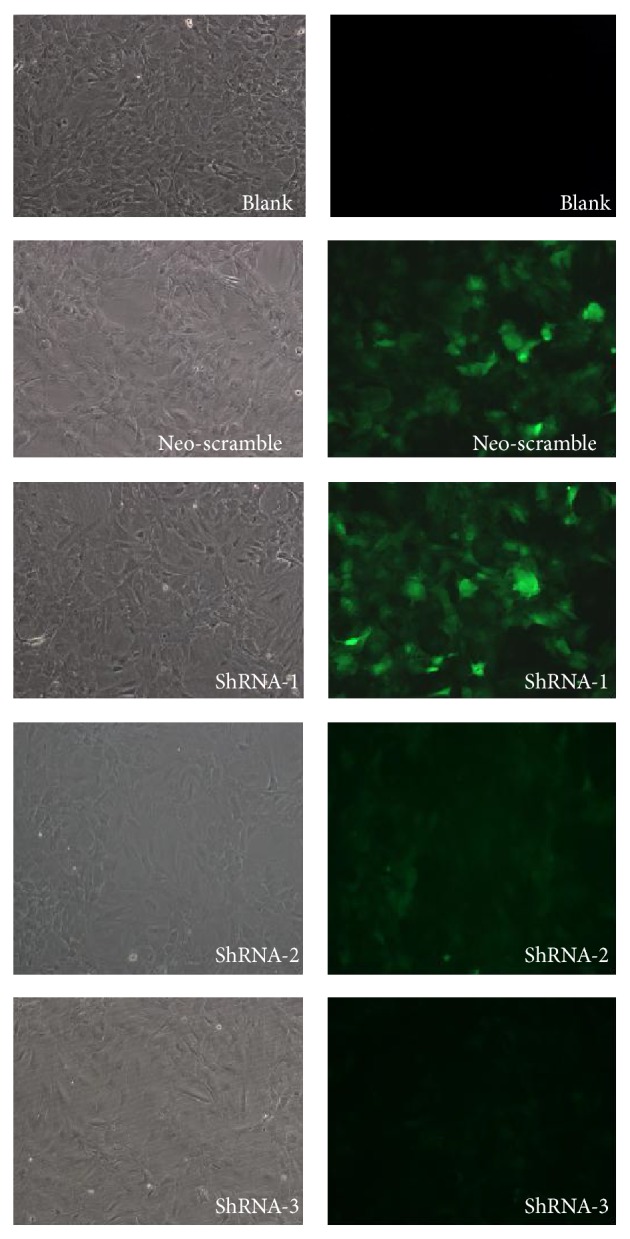
Infection efficiency in cells transfected with different lentiviral vectors. The NS and ShRNA-1 group had infection efficiency at approximately 70%, shRNA-2 group had an infection efficiency of 45%, and the shRNA-3 group had an infection efficiency of 20%.

*(1) Determination of Lentiviral Infection Efficiency*. Lentiviral infection efficiency was determined by calculating the percentage of fluorescent cells in 10 randomly selected high power fields. The NS and shRNA-1 group had infection efficiency at approximately 70%, shRNA-2 group had an infection efficiency of 45%, and the shRNA-3 group had an infection efficiency of 20% (Figure 3).

**Figure 4:**
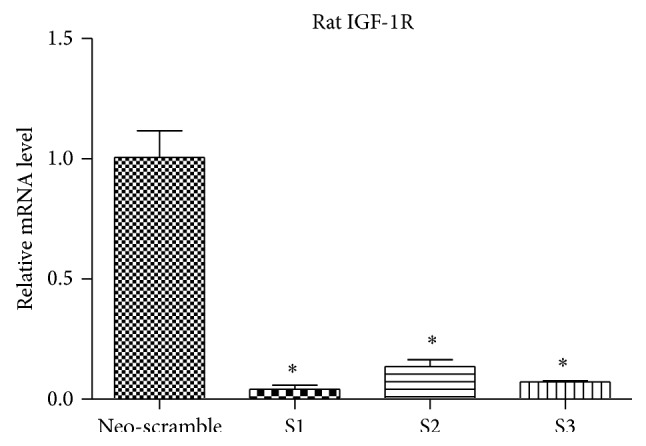
Effects of shIGF-1R transfection on IGF-1R expression in osteoblasts. ^*^ *P* < 0.01 compared with NS group. The experiment was repeated 3 times.

*(2) Selection of the Optimal Interference Sequence*. Cells were, respectively, transfected with shRNA-1 (S1), shRNA-2 (S2), shRNA-3 (S3), and NS vector (NS), and RT-PCR was performed to detect IGF-1R expression after 72 h of cell culturing. Compared with the control group, cells in S1, S2, and S3 all showed significantly silenced IGF-1R expression, and the S1 group exhibited the highest level of IGF-1R silencing (Figure 4).

### 3.4. Detection of Cell Proliferation

**Figure 5:**
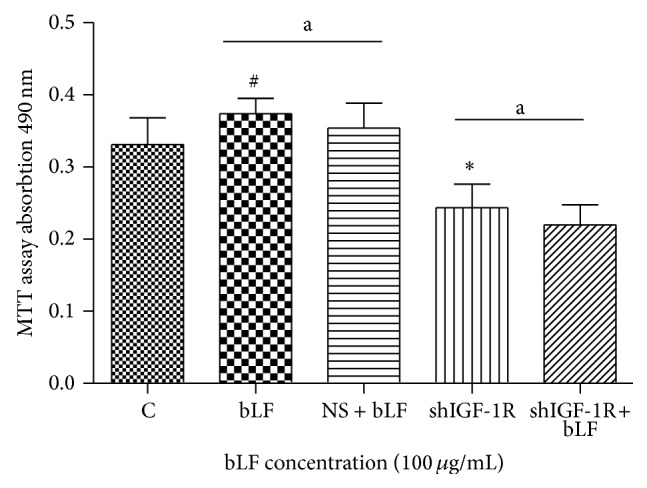
Effects of lactoferrin in shIGF-1R transfected osteoblast proliferation. ^*^ *P* < 0.01 and ^#^ *P* < 0.05 compared with the control. ^a^ *P* > 0.05 compared between bLF and NS + bLF group and compared between shIGF-1R and shIGF-1R+bLF group. NS: Neo-scramble (empty vector).

MTT-staining revealed that lactoferrin concentrated at 100 *μ*g/mL significantly promoted osteoblast proliferation, while shIGF-1R silencing significantly suppressed osteoblast proliferation. No significant difference in cell proliferation was detected between shIGF-1R cells treated with and without 100 *μ*g/mL lactoferrin (Figure 5).

### 3.5. Detection of Cell Apoptosis

**Figure 6:**
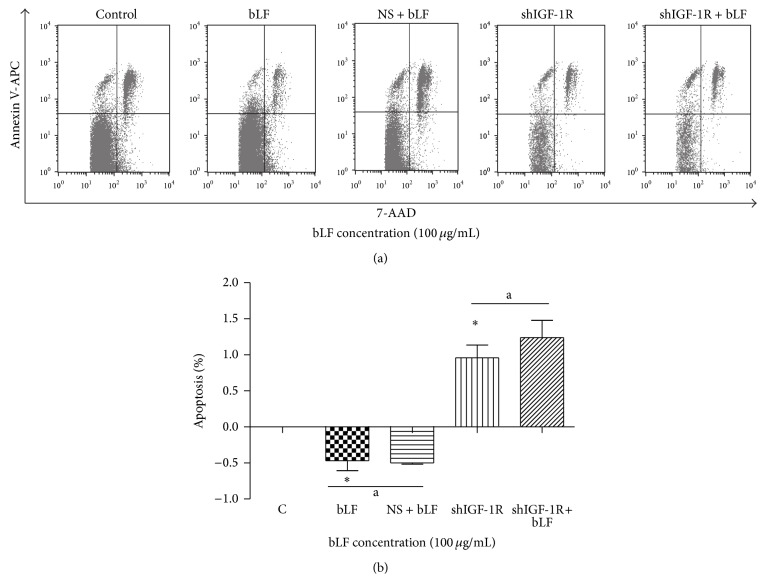
Effects of lactoferrin in shIGF-1R transfected osteoblast apoptosis. ^*^ *P* < 0.01 compared with the control. ^a^ *P* > 0.05 compared between bLF and NS + bLF group, and compared between shIGF-1R and shIGF-1R+bLF group. NS: Neo-scramble (empty vector).

Flow cytometric analysis indicated that lactoferrin concentrated at 100 *μ*g/mL significantly inhibited osteoblast apoptosis, while shIGF-1R silencing significantly promoted osteoblast apoptosis. No significant difference in cell apoptosis was detected between shIGF-1R cells treated with and without 100 *μ*g/mL lactoferrin (Figure 6).

### 3.6. Detection of IGF-1R Downstream Signaling in Osteoblast

**Figure 7:**
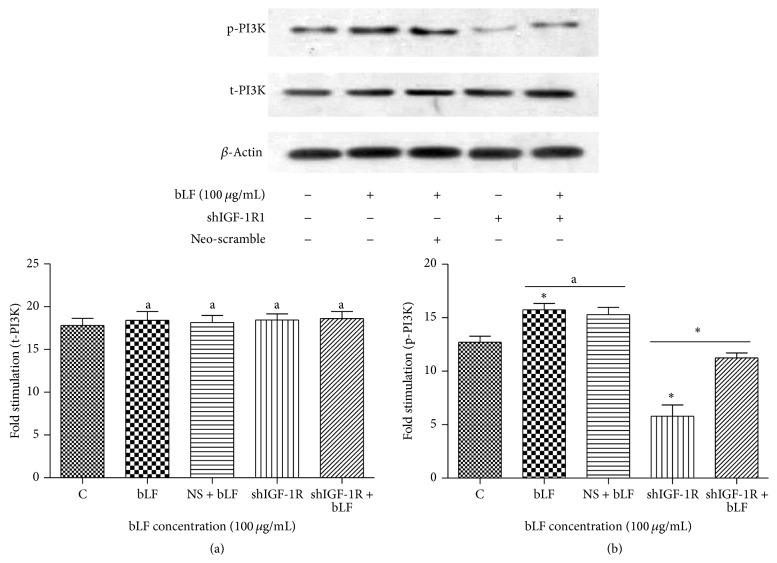
Effects of lactoferrin on PI3K phosphorylation in shIGF-1R transfected cells. ^*^ *P* < 0.01 compared with the control. ^a^ *P* > 0.05 compared between bLF and NS + bLF group. ^*^ *P* < 0.01 compared between shIGF-1R and shIGF-1R+bLF group. NS: Neo-scramble (empty vector).

Western blot suggested that when compared with the control, lactoferrin intervention at a concentration of 100 *μ*g/mL significantly induced the expression of p-PI3K and p-RAS, while shIGF-1R silencing significantly decreased the expression level of p-PI3K and p-RAS. Lactoferrin-treated shIGF-1R cells exhibited significantly increased level of p-PI3K and p-RAS when compared with shIGF-1R cells (Figure 7).

## 4. Discussion

Our previous study confirmed that after different days of intervention (1 d, 3 d, 5 d, and 7 d), differently concentrated lactoferrin (0.1 *μ*g/mL, 1 *μ*g/mL, 10 *μ*g/mL, 100 *μ*g/mL, and 1000 *μ*g/mL) could induce osteoblast proliferation and differentiation and could also inhibit osteoblast apoptosis and death. We have identified the optical concentration (100 *μ*g/mL) and time (5 d) for lactoferrin intervention [7]. However, the molecular mechanism by which lactoferrin induced osteoblast proliferation was yet to be clear. This study suggested that lactoferrin induced the expression of IGF-1 mRNA and protein in a concentration-dependent manner. Furthermore, as the osteoblasts expressed IGF-1R, a receptor involved in regulating cell proliferation, differentiation, and apoptosis, the lactoferrin could exert a concentration-dependent effect in promoting IGF-1R transcription and expression. By silencing the expression of IGF-1R and by detecting the level of IGF-1R downstream signaling pathway, we investigated the relationship between lactoferrin, IGF-1R, and osteoblast proliferation and apoptosis and explained the molecular mechanism of lactoferrin in inducing osteoblast proliferation.

Lactoferrin (80 kDa) is an iron-binding glycoprotein belonging to the transferrin family, and it mainly exists in breast milk, epithelial secretion, and neutrophil secretory vesicles. Lactoferrin is a multieffect factor involved in antibacteria and immunomodulation, and more importantly, it can induce osteoblast proliferation and differentiation, while inhibiting osteoblast apoptosis and osteoclastogenesis [8, 9]. In our previous study, we found that lactoferrin increased bone mineral density, improved bone microstructure, promoted bone formation, and inhibited bone resorption in ovariectomized rats [3].* In vitro* study also showed that lactoferrin intervention (100 *μ*g/mL) for consecutive 5 d significantly induced osteoblast proliferation [7]. When compared with the control, osteoblast received 24 h lactoferrin intervention displayed significantly suppressed serum deprivation-induced apoptosis [10].

Grey et al. [9] detected the express of LRP1 and LRP2 on osteoblast cell surface. Since lactoferrin could activate the P42/44/MAPK pathway via LRP1, their finding indicated that LRP1 at least partially participated in the regulation of osteoblast proliferation. Lactoferrin could also induce cell proliferation by activating PI3K, but receptors for such signaling transduction were yet to be identified. On the other hand, Grey et al. [9] demonstrated that lactoferrin inhibited osteoblast apoptosis through a LRP1-independent pathway, but the molecules involved in this pathway, and the mechanisms by which lactoferrin inhibited cell apoptosis were still unclear.

The IGF-1R is a tetramer consisting of 2 *α* subunits and 2 *β* subunits. After binding to its ligand (IGFl/IGF2), IGF-1R could, via the mediation of IGFBPs, significantly induce mitosis, enhance DNA synthesis, and promote cell proliferation and differentiation of the target cells [11, 12]. IGF-1R is an important member of the tyrosine kinase receptor family. Binding of IGF-1 to IGF-1R would induce the phosphorylation of insulin receptor substrate-1 (IRS-1), thereby activating the downstream signaling pathways [13]. The PI3K-dependent AKT pathway and the RAS-mediated MAPK pathway are 2 pathways that are more intensively studied regarding the IGF-1R-regulated downstream signaling pathways [14]. In this study, we designed 3 interfering sequences for IGF-1R (S1, S2, and S3), constructed IGF-1R lentiviral vectors, selected the optimal interfering sequence S1 using the RT-PCR technique, and silenced the expression of IGF-1R by transfecting the recombinant vector into osteoblasts. Compared with the control, osteoblasts with silenced IGF-1R exhibited significantly decreased proliferation while increasing apoptosis, indicating that IGF-1R is an essential receptor enabling osteoblasts to maintain normal mitosis and avoid apoptosis. In order to clarify whether the lactoferrin-induced osteoblast proliferation and apoptosis inhibition were mediated by IGF-1R, we performed lactoferrin intervention (100 *μ*g/mL) in IGF-1R-silenced osteoblasts. Lactoferrin significantly induced osteoblast proliferation and suppressed cell apoptosis, indicating that the function of lactoferrin was IGF-1R-dependent.

**Figure 8:**
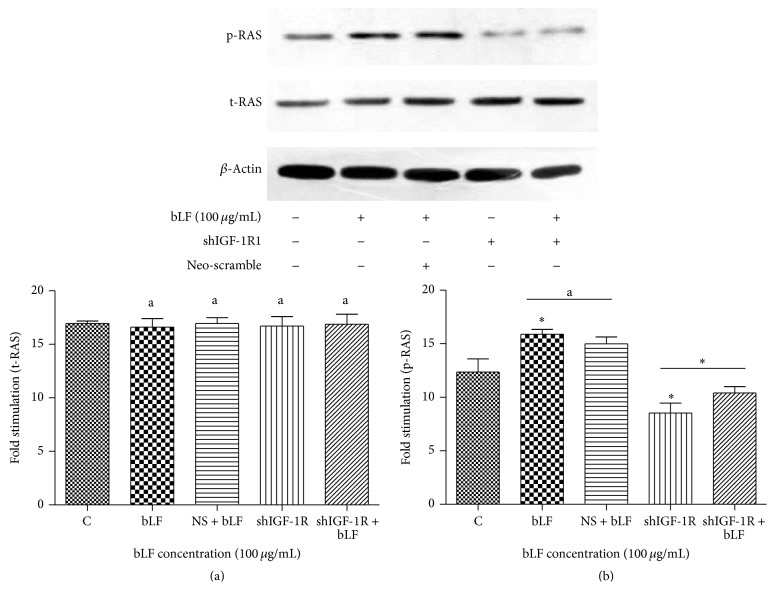
Effects of lactoferrin on RAS phosphorylation in shIGF-1R transfected cells. ^*^ *P* < 0.01 compared with the control. ^a^ *P* > 0.05 compared between bLF and NS + bLF group. ^*^ *P* < 0.01 compared between shIGF-1R and shIGF-1R+bLF group. NS: Neo-scramble (empty vector).

Western blot revealed that lactoferrin could activate the phosphorylation of both PI3K and RAS; thus, the lactoferrin-triggered IGF-1R downstream pathway mainly involved the PI3K-dependent AKT pathway and the RAS-dependent MAPK pathway. When compared with the control, IGF-1R-silenced cells displayed significantly decreased level of PI3K and RAS phosphorylation, while the intervention of lactoferrin (5 d, 100 *μ*g/mL) elevated PI3K and RAS phosphorylation, and the difference was statistically significant. This result suggested that, in IGF-1R knockout osteoblasts, lactoferrin could still activate PI3K and RAS phosphorylation, and such activation might be mediated through an IGF-1R-independent pathway (Figure 8).

Among the various lactoferrin receptors, LRP1 and LRP2 are 2 receptors expressed on osteoblast cell surface and are multiple-ligand members of the LRP family [6]. In osteoblast, functional LRP1 could mediate the endocytosis of lactoferrin and induce the formation of cytoplasmic membrane-bound vesicles. As a receptor involved in mitogenic signaling, LRP1 could also activate the p42/44 mitogen-activated protein kinase (MAPK) pathway, thereby inducing the mitosis of osteoblasts. These findings suggested that LRP1 at least partially participated in the lactoferrin-induced mitosis in osteoblasts. In addition, lactoferrin could also activate the PI3K-dependent Akt pathway in a LRP1-independent manner [15], but the mechanism requires further studies.

Grey et al. [9] have confirmed that LRP1 could induce osteoblast mitosis by phosphorylating MAPK, and we observed that the MAPK pathway was activated, via LRP1, in osteoblast with silenced IGF-1R expression. However, the receptors involved in the lactoferrin-induced PI3K-dependent Akt activation were yet to be identified. The findings of our study demonstrated that IGF-1R was not a key receptor mediating the lactoferrin-dependent PI3K and RAS-dependent MAPK pathway activation, and whether lactoferrin could activate PI3K signaling through other receptors would need more investigation. Fulzele et al. [11] found that silence of IGF-1R induced insulin receptor (IR) upregulation. Since the PI3K/AKT and RAS/MAPK are the major pathways regulating IR downstream signaling transduction; whether lactoferrin activates PI3K via IR requires further studies.

In conclusion, we found that lactoferrin induced IGF-1/IGF-1R expression in a concentration dependent manner, and it induced proliferation while inhibiting apoptosis of osteoblasts through the mediation of IGF-1R. We also identified that lactoferrin activated the PI3K/RAS signaling pathway through an IGF-1R-independent mechanism.

## Figures and Tables

**Table 1:** Primer sequences.

Gene	Genbank numbers	Primer sequence	Product size (bp)
*β*-Actin	NM-031144.2	Forward 5′-GGAGATTACTGCCCTGGCTCTA-3′	150
		Reverse 5′-GACTCATCGTACTCCTGCTTGCTG-3′	

IGF-1	NM 001082478.1	Forward 5′-GCACTCTGCTTGCTCACCTTTA-3′	148
		Reverse 5′-TCCGAATGCTGGAGCCATA-3′	

IGF-1R	NM 052807.2	Forward 5′-GGTCTCTAAGGCCAGAGGTGGA-3′	122
		Reverse 5′-GACGAACTTGTTGGCATTGAGGTA-3′	

**Table 2:** IGF-1R shRNA sequences.

Name	Sequence
S1	Forward: CCGGGCGGTGTCCAATAACTACATTCTCGAGAATGTAGTTATTGGACACCGCTTTTTG
	Reverse: AATTCAAAAAGCGGTGTCCAATAACTACATTCTCGAGAATGTAGTTATTGGACACCGC

S2	Forward: CGGCCAACGAGCAAGTTCTTCGTTCTCGAGAACGAAGAACTTGCTCGTTGGTTTTTG
	Reverse: ATTCAAAAACCAACGAGCAAGTTCTTCGTTCTCGAGAACGAAGAACTTGCTCGTTGG

S3	Forward: CCGGAGCAGGTTGTAACAATCTATTCTCGAGAATAGATTGTTACAACCTGCTTTTTTG
	Reverse: AATTCAAAAAAGCAGGTTGTAACAATCTATTCTCGAGAATAGATTGTTACAACCTGCT
